# Automated Quantification of Macular Ellipsoid Zone Intensity in Glaucoma Patients: the Method and its Comparison with Manual Quantification

**DOI:** 10.1038/s41598-019-56337-7

**Published:** 2019-12-24

**Authors:** Ahnul Ha, Sukkyu Sun, Young Kook Kim, Jin Wook Jeoung, Hee Chan Kim, Ki Ho Park

**Affiliations:** 1 Department of Ophthalmology, Seoul National University College of Medicine, Seoul, Korea; 2 Department of Ophthalmology, Seoul National University Hospital, Seoul, Korea; 3 Interdisciplinary Program, Bioengineering Major, Graduate School, Seoul National University, Seoul, Korea; 4 Seoul Glaucoma Image Post-processing Laboratory (SGIP LAB), Seoul, Korea; 5 Department of Biomedical Engineering, Medical Research Center, Seoul National University College of Medicine, Seoul, Korea

**Keywords:** Eye diseases, Medical imaging

## Abstract

The macular ellipsoid zone intensity (mEZi) is a known marker of disease severity in a number of diverse ocular diseases. The purpose of this study was to establish an automated method (AM) for mEZi quantification and to compare the method’s performance with that of a manual method (MM) for glaucoma patients and healthy controls. Seventy-one (71) mild-to-moderate glaucoma patients, 71 severe-glaucoma patients, and 51 controls were enrolled. Both calibration (*n* = 160) and validation (*n* = 33) image sets were compiled. The correlation of AM to MM quantification was assessed by Deming regression for the calibration set, and a compensation formula was generated. Then, for each image in the validation set, the compensated AM quantification was compared with the mean of five repetitive MM quantifications. The AM quantification of the calibration set was found to be linearly correlated with MM in the normal-to-severe-stage glaucoma patients (*R*^2^ = 0.914). The validation set’s compensated AM quantification produced *R*^2^ = 0.991, and the relationship between the 2 quantifications was AM = 1.004(MM) + 0.139. In the validation set, the compensated AM quantification fell within MM quantification’s 95% confidence interval in 96.9% of the images. An AM for mEZi quantification was calibrated and validated relative to MM quantification for both glaucoma patients and healthy controls.

## Introduction

Glaucoma manifests as pathological changes to the retinal ganglion cells (RGCs) of the inner retina^1,2^. It has been noted that degenerative changes within the lateral geniculate nucleus and visual cortex can, by trans-synaptic degeneration, co-present in glaucomatous eyes, and that they do so in relation to the severity of RGC cell loss^3^. The mechanisms underlying these processes remain unclear, but, based on the close structural and functional relationship of retinal neuronal cells, it has been hypothesized that outer-retinal-layer changes might also occur, according to similar principles. In fact, impaired outer-retinal neuronal function along with cell-number reduction in the photoreceptor layer has been demonstrated for glaucomatous eyes^4–7^.

The outer retina’s second hyper-reflective band on spectral-domain optical coherence tomography (SD-OCT) imaging is a known marker of severity in a number of diverse retinal diseases including, but not limited to, age-related macular degeneration and inflammatory disease^8–11^. Whereas this band traditionally has been associated with the inner/outer segment junction of photoreceptors, more recent studies indicate a correlation anatomically with the inner-segment ellipsoid, which is referred to internationally as the ellipsoid zone (EZ)^12,13^. The EZ, densely packed with mitochondria, is essential to the structural integrity and function of photoreceptors; and fulfills important metabolic and light-guiding roles^14,15^. Therefore, observation of glaucoma patients’ EZ changes can possibly provide clues to involvement of the outer retina in cases of glaucoma.

Gin *et al*. suggested a quantitiative method for measurement and analysis of EZ band intensity on SD-OCT^10^. Recently, our group reported cases of relative macular EZ intensity (mEZi) reduction on SD-OCT for the mild-to-moderate and severe glaucoma stages, having determined that the extent of reduction was positively correlated with glaucoma severity^16^. These findings suggest, though tentatively, that secondary changes to mitochondria packed tightly within the inner-segment ellipsoids of photoreceptors might be incurred in the course of glaucoma progression.

The following sections of this paper explain the methodological approach of our automated method (AM)’s software application for quantification of mEZi and demonstrate its results. In addition, its performance for glaucoma patients and healthy controls is compared with that of a manual method (MM).

## Results

The Table 1 reports the mean ± standard deviation (SD) along with the mEZi quantification range by each method for the three image sets (normal, mild-to-moderate glaucoma, severe glaucoma) used in the calibration and validation studies, respectively. These data confirmed the fact that the normal and glaucoma groups represented a range of mEZi and that those ranges were similar between the two methods.

**Table 1:** Macular Ellipsoid Zone Intensity within the Normal, Mild-to-Moderate, and Severe Glaucoma Stage in the Calibration and Validation Studies by Quantification Method.

	Calibration Study *(n* = *160)*				Validation Study *(n* = *33)*					
	MM		AM		MM*		AM		AM^†^	
	Mean ± SD	Range	Mean ± SD	Range	Mean ± SD	Range	Mean ± SD	Range	Mean ± SD	Range
Normal	**4.08** ± **0.42**	3.32–5.19	**4.15** ± **0.55**	3.10–5.18	**4.12** ± **0.45**	3.75–5.08	**4.46** ± **0.44**	3.96–5.32	**4.31** ± **0.42**	3.84–5.13
Mild-to-moderate Glaucoma	**3.14** ± **0.43**	2.21–4.48	**3.21** ± **0.51**	2.06–4.70	**3.13** ± **0.38**	2.78–4.08	**3.31** ± **0.38**	2.93–4.31	**3.22** ± **0.36**	2.86–4.17
Severe Glaucoma	**2.36** ± **0.52**	1.01–3.40	**2.47** ± **0.56**	1.02–3.81	**2.36** ± **0.28**	1.89–2.87	**2.60** ± **0.29**	2.13–3.07	**2.54** ± **0.28**	2.09–2.99

*MM quantification in the validation study are the mean of five separate counting sessions.

^†^AM quantification in the validation study are compensated using the equation for the regression line generated in the calibration study.

### Calibration of AM versus MM mEZi Quantification

**Figure 1:**
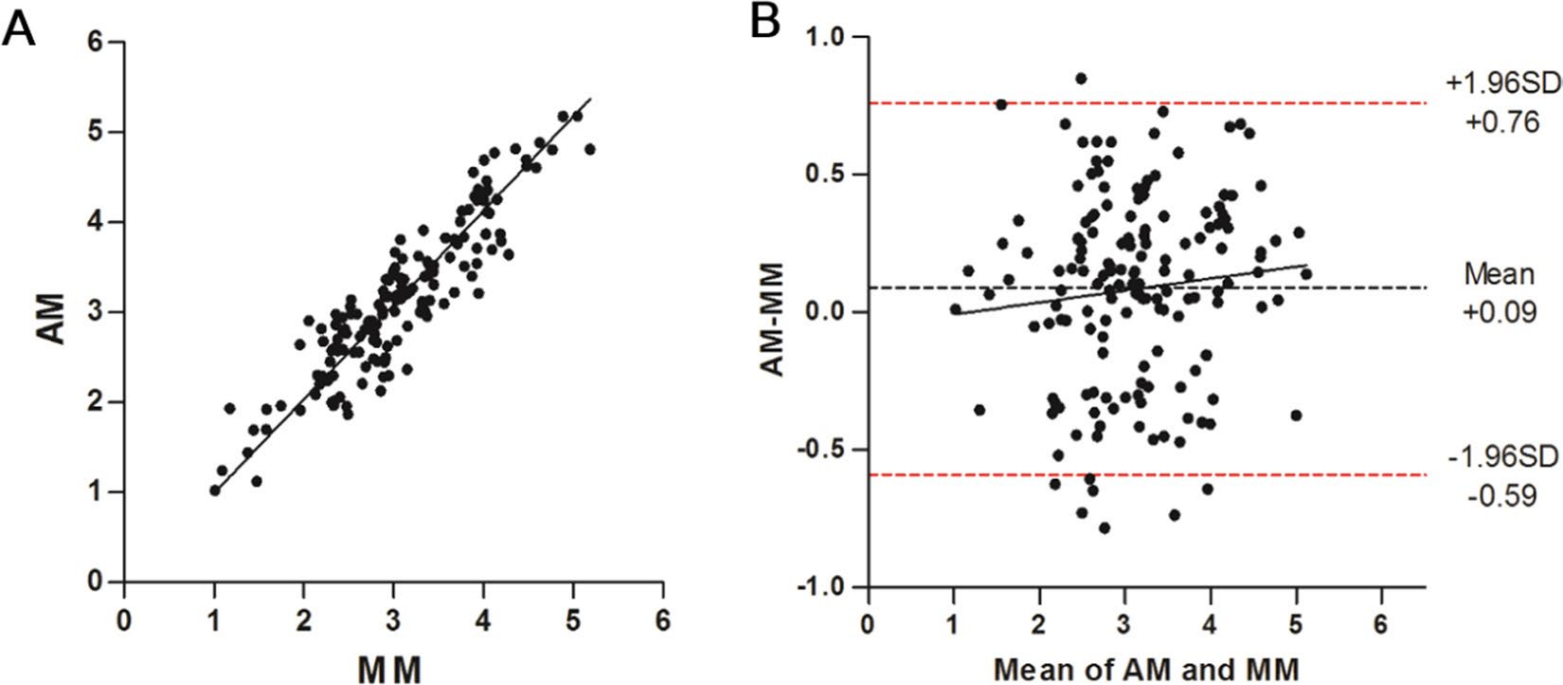
Automated method (AM) versus manual method (MM) of mEZi quantification with 160 images. (**A**) The correlation produced an *R*^2^ = 0.914 (*P* < 0.001). The relation of AM-to-MM quantification, as established by Deming regression, was defined by the solid line according to the formula AM = 1.047(MM) − 0.058. (**B**) Bland-Altman plot for 160 calibration images. The dashed red lines represent the 95% limits of agreement between the AM and MM (−0.59 and + 0.76).

The AM versus MM quantifications are plotted for all 160 images sets in Fig. 1A. The correlation produced *R*^2^ = 0.914 (*P* < 0.001). The relationship between the AM (abscissa) and MM quantifications (ordinate) was AM = 1.047(MM) − 0.058, as established by Deming regression. These data indicated that there was a close correlation between the AM and MM quantifications for the normal eyes and also through the entire mild-to-moderate-to-severe range of glaucomatous damage. A Bland-Altman plot (Fig. 1B) showed an average difference of 0.09 mEZi (AM > MM), which increased slightly with mEZi. The limit-of-agreement range was −0.59–+0.76 mEZi.

### Validation of AM versus MM Quantification

**Figure 2:**
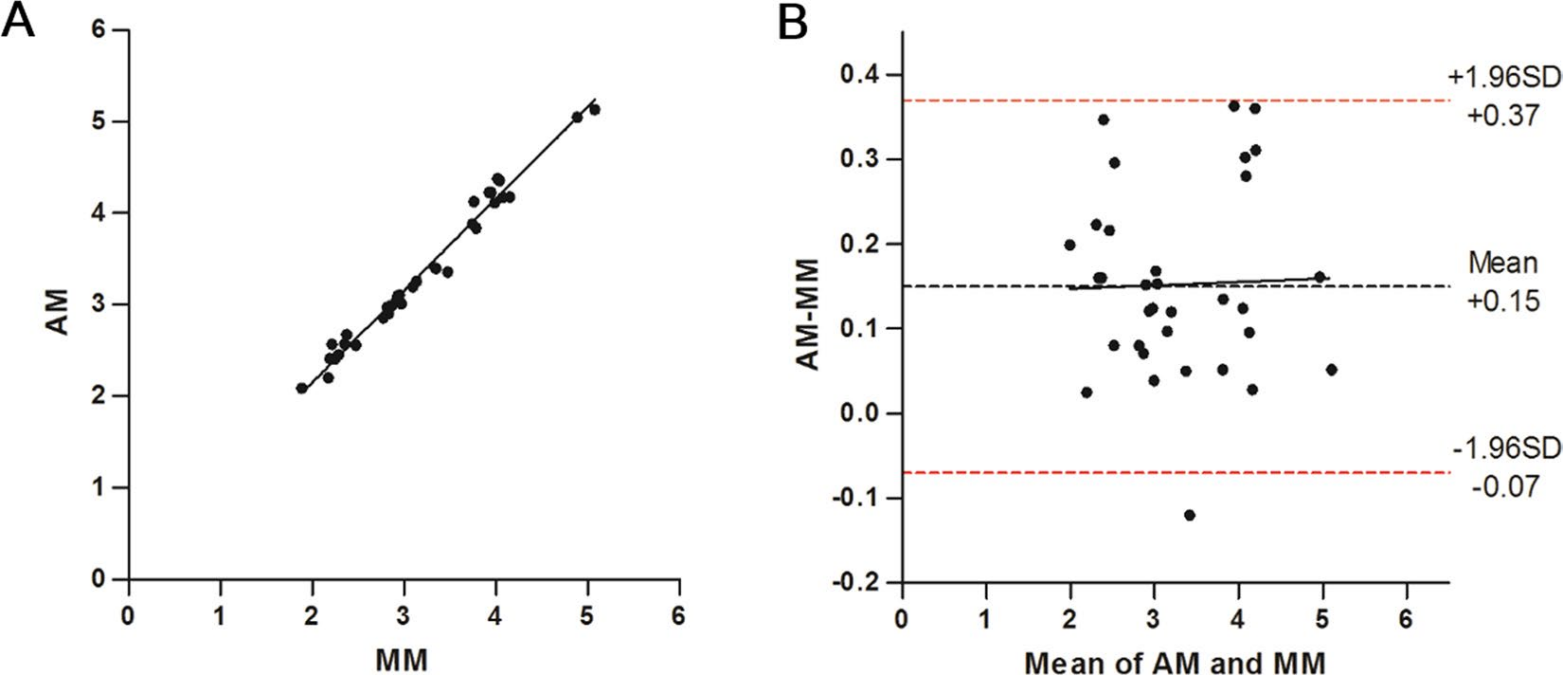
Validation of calibration equation between AM and mean of five MM mEZi quantifications for a second group of 33 images. (**A**) AM quantifications were compensated by the regression line equation that had been established in the initial calibration study, and were then compared with the mean of five repeated MM quantifications for each image. The solid line represents the Deming regression result: AM = 1.004(MM) + 0.139, *R*^2^ = 0.991. (**B**) Bland-Altman plot for 33 validation images. The dashed red lines represent the 95% limits of agreement between the AM and MM (−0.07 and + 0.37).

The distribution of mEZi (by MM) in validation study was comparable with that of calibration study. The mean ± SD of MM quantification in the calibration set (4.08 ± 0.42 in normal group; 3.14 ± 0.43 in mild-to-moderate glaucoma group; 2.36 ± 0.52 in severe glaucoma group) was not significantly different from the mean ± SD of MM quantification (4.12 ± 0.45 in normal group; 3.13 ± 0.38 in mild-to-moderate glaucoma group; 2.36 ± 0.28 in severe glaucoma group) used in the present validation set (Mann-Whitney test [*P* = 0.991, 0.893 and 0.446, respectively]). In Fig. 2A, the compensated AM quantification is plotted versus the mean of the five MM quantifications for each of the 33 image sets. The correlation produced *R*^2^ = 0.991 (*P* < 0.001). The relationship between the AM (abscissa) and MM quantifications (ordinate) was AM = 1.004(MM) + 0.139, as established by Deming regression. Figure 2B shows the Bland-Altman plot, with an average difference of 0.15 mEZi (AM > MM), the limits of agreement extending from −0.07 to + 0.37. The AM quantification fell within the 95% CI of MM counts for 32 of the 33 images sets (96.9%).

### Comparison of AM versus MM mEZi Quantification for Different Stages of Glaucoma

Supplementary Fig. S1 shows AM versus MM quantifications in mild-to-moderate glaucoma group and severe glaucoma group. By Deming regression, the relationship between the AM (abscissa) and MM quantifications (ordinate) was AM = 1.239(MM) − 0.680 in the mild-to-moderate glaucoma group and AM = 1.099(MM) − 0.128 in the severe glaucoma group, with 120 image sets for calibration. For the 22 validation sets, the Deming regression showed AM = 0.955(MM) + 0.222 in the mild-to-moderate glaucoma group and AM = 0.976(MM) + 0.238 in the severe glaucoma group. The Bland-Altman plots demonstrated that both the differences and the limits of agreement of AM versus MM mEZi quantification were similar between the mild-to-moderate glaucoma group and the severe glaucoma group.

### Automated mEZi map

**Figure 3:**
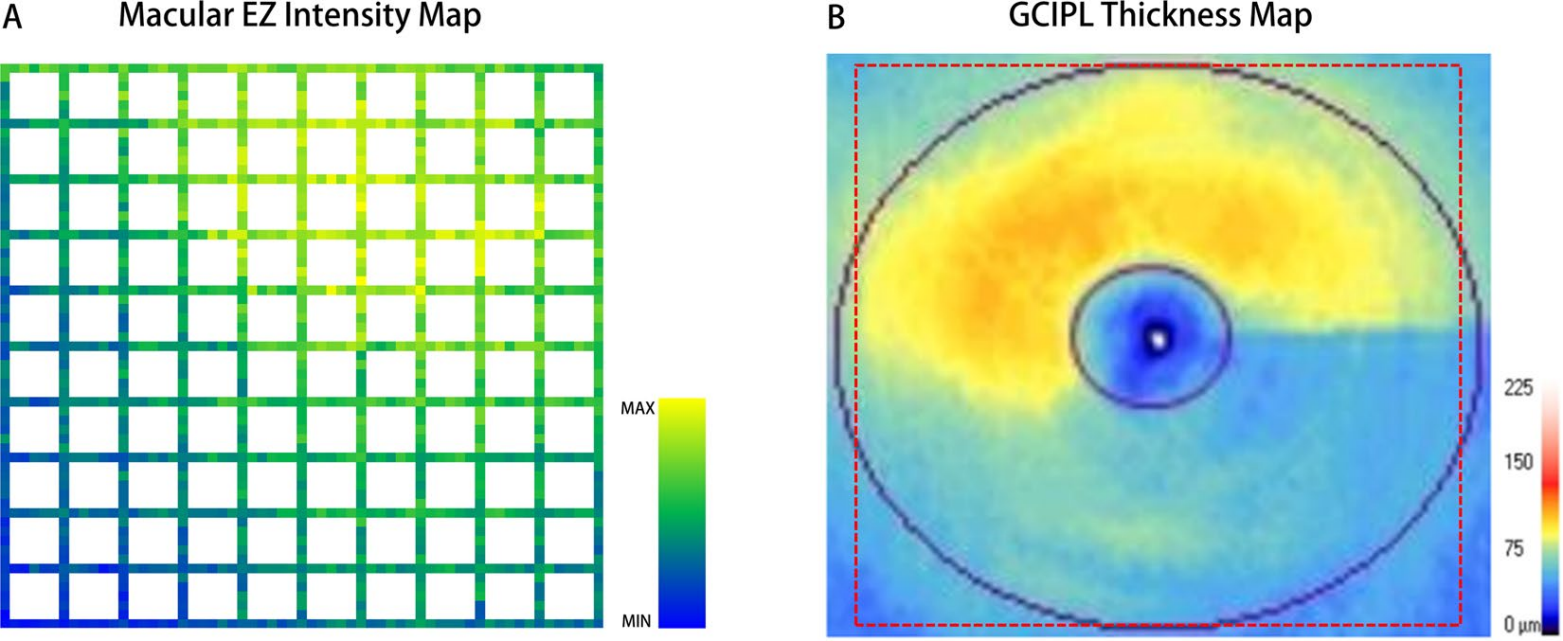
Representative mEZi quantification results by AM. The patient was a 56-year-old male with primary open-angle glaucoma (POAG) in the mild-to-moderate stage (visual field mean deviation = −4.6 dB). (**A**) Color map indicating mEZi in each location of left eye. The greater mEZi values are yellow-colored, and the smaller values are blue-colored. (**B**) Macular ganglion cell–inner plexiform layer (GCIPL) thickness map on cirrus OCT showing inferior GCIPL defect. The dotted red box indicates the area covered by the mEZi color map.

Figure 3 provides representative AM-based mEZi quantification cases. The patient was a 56-year-old male with primary open-angle glaucoma of the mild-to-moderate stage (VF MD = −4.6 dB) in his left eye. The macular ganglion cell–inner plexiform layer (GCIPL) thickness map, on cirrus OCT, showed inferior GCIPL defect. A mEZi color map indicating the mEZi value for each location was generated, the inferior macula showing reduced mEZi relative to the superior region. The greater mEZi values are yellow-colored, while the smaller values are blue-colored.

The purpose of this paper was to present an automated algorithm and software application for mEZi quantification and to assess the method’s performance relative to a previously published manual method with SD-OCT images taken from normal and glaucomatous eyes of differing disease stages. The proposed method’s results proved to be highly correlated to those of manual mEZi measurement for an initial calibration of 160 images. It also showed a performance similar to that of an experienced ophthalmologist for a validation set of 33 images counted five times each, its results falling within the ophthalmologist’s 95% CI for 32 of the 33 images. Since both sets of images demonstrated mEZi quantification that ranged from normal to end-stage glaucoma, the obtained results indicated that the proposed method met the requirements laid out at the beginning of the work: an automated mEZi calculator that is applicable to all levels of glaucomatous damage as well as computationally robust and efficient.

In specific performance, the AM took less than 1 minute to obtain a final mEZi value for one SD-OCT image, according to the test system used in the present study (MacBook Pro laptop, 2.2 GHz Core2 Duo, 3GB RAM; Apple, Cupertino, CA, USA), at least. Meanwhile, manual mEZi calculation took at least 5 to 10 minutes for complete analysis of the same image. Thus, analysis of a large number of images or a wide range of areas by the MM can be time consuming and prone to error. We anticipate that the proposed automated mEZi analysis method will find application to larger and more diverse datasets and will foster additional research of photoreceptor change in glaucoma as well as further evaluation of its clinical significance.

It should be noted that the application of the method proposed in this paper can be expanded to other diseases. EZ intensity is known to be a marker of severity in many diverse retinal pathologies including, among others, age-related macular degeneration and inflammatory diseases^8–11^. Due to disruption of the outer retinal layer by lesions such as drusen, reticular pseudodrusen, or hyperreflective foci in retinal diseases^10^, however, the overall analysis protocol might require modification along with further validation. Additionally, AM analysis is easily modifiable for quantification of the intensities of outer retinal layers other than the EZ, or for calculation of intensities using other retinal layers as reference values. The process of expanding the software in these directions is already underway.

**Figure 4:**
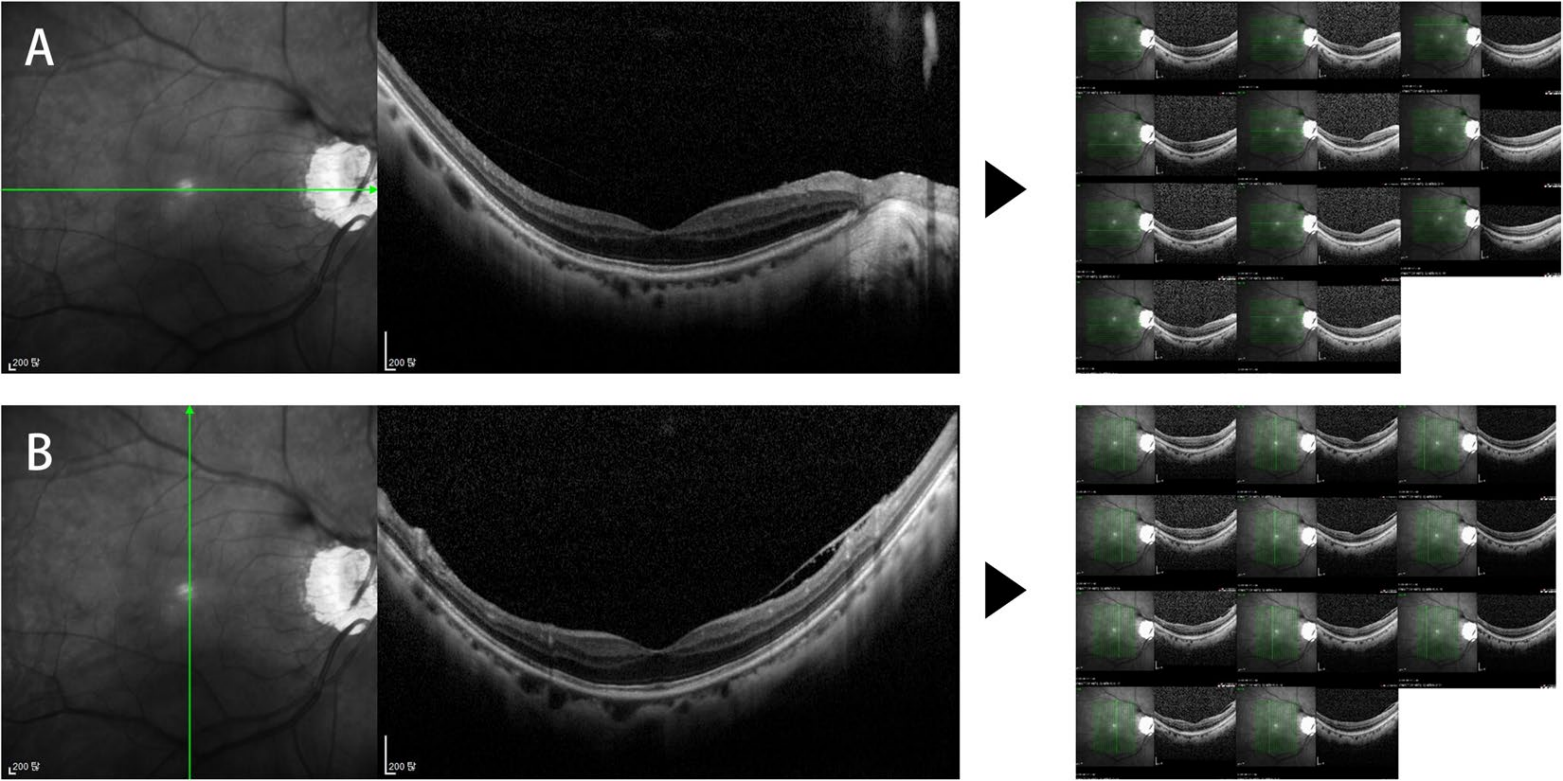
Examples of optical coherence tomography (OCT) macular scans for which AM failed to quantify mEZi. (**A**) Horizontal line scans of 60-year-old female POAG patient with myopia (spherical equivalent, −5.25 diopters; axial length, 25.3 mm). (**B**) Vertical line scans of same patient. In this eye with a highly curved posterior structure, the AM often failed to crop the area of interest.

The present study’s findings should be interpreted in the light of its limitations. First, the sampling number differed between the two methods: each meridian consisted of 20 retinal segments and one central segment in the MM, but there was a total of 220 samples for the AM. However, EZ intensity reduction during glaucoma-stage advancement has been proposed to occur in the form of an overall retinal change rather than as a focal pathologic change^16^. Thus, the two methods’ average values would have shown, regardless of the number of analysis samples, high agreement. Second, mEZi quantification by the AM showed a failure rate of 6.28%. In the OCT scans, the retinal layers were not always parallel to the image’s horizontal plane, and moreover, had an irregularly curved shape. In eyes with a highly curved posterior structure, the AM often failed to crop the area of interest. Figure 4 shows an example of OCT scans for which the AM failed to quantify mEZi. Future modifications of the AM to minimize the rate of analysis failure are planned. Third, whereas compensation of automated mEZi quantification was required for the fairest comparison of the AM performance with the MM’s in the validation study, it is not believed that automated counts should be compensated in actual scientific applications. Doing so would imply that the manual mEZi analysis method is actually the gold standard, and thus, that it is more accurate than the present study’s AM, which is difficult, if not impossible, to determine at this point. Accordingly, future research evaluating the accuracies of the two methods by comparison of molecular changes in photoreceptors will be necessary. Fourth, the performance of the described methods depends, as for any other image processing algorithm, on the quality of the input images. We were careful to include only OCT images with no artifacts and good image quality. Our results might not be directly applicable to lower-quality OCT images obtained in an actual clinical setting, therefore.

In summary, this study presented a novel method for automated mEZi quantification based on SD-OCT images. The calibration and validation data indicate that its results are similar to those of manual mEZi calculation through a wide range — normal to end-stage — of glaucomatous damage. We expect that this automated mEZi quantification method will find application in various studies in the field of ophthalmology.

## Methods

This study was approved by the Seoul National University Hospital Institutional Review Board and faithfully adhered to the tenets of the Declaration of Helsinki. All participants provided their written informed consent.

### Overview of study design

First, a calibration study was performed in order to compare mEZi for the two methods (AM *vs*. MM) with an initial group of 160 image sets. These images had been obtained from normal (*n* = 40), mild-to-moderate glaucoma (*n* = 60), and severe glaucoma (*n* = 60) eyes so as to achieve a balance among the damage levels. Next, a validation study was conducted for comparison of AM with MM quantification, specifically by determining whether or not AM quantification fell within the range of repeated MM quantifications. For this, MM quantification was repeated five times for a separate group of 33 image sets representing a similarly broad range of glaucomatous damage (11 images per group). The AM quantification was compared with both the mean MM quantification and the range of MM re-quantification variability (95% confidence interval [CI]). Finally, in order to demonstrate the overall automated mEZi quantification strategy, SD-OCT image acquisition, AM mEZi measurement, and AM mEZi topographic maps were generated for normal and glaucomatous eyes.

### Study subjects

All of the study subjects had been examined between January 2015 and December 2018 at the Seoul National University Hospital Glaucoma Clinic in Seoul, Korea.

For inclusion in the study, subjects had to be 40 to 65 years old, have a spherical refraction that was greater than −6 diopters (D) and less than 3 D, an open anterior chamber angle, and show reliable visual field (VF) test results. The exclusion criteria were: (1) a history of intraocular surgery (except uncomplicated cataract surgery) or retinal laser photocoagulation; (2) any neurological and/or systemic diseases potentially affecting the retinal structure and/or function or VF results. Additionally, any cases of suspicious retinal lesions potentially affecting the outer retinal layer, such as in cases of inflammatory conditions or hereditary or degenerative retinal diseases, were excluded.

Glaucomatous eyes were defined based on the characteristic optic disc appearance (localized or diffuse neuroretinal rim thinning/notching) on stereo disc photography (SDP), red-free fundus imaging of the presence of retinal nerve fiber layer (RNFL) defect in the corresponding region, and the presence of VF defect corresponding to structural change. Optic disc signs on SDP and RNFL changes on red-free imaging were independently evaluated by two glaucoma specialists (AH and KHP) masked to all non-relevant clinical data. Discrepancies between them were resolved by consensus. Based on a reliable (false-positives/negatives <15%, fixation losses <15%) Humphrey Visual Field (HVF) result obtained within 3 months of SD-OCT imaging, the glaucoma patients were divided into two groups: mild-to-moderate glaucoma (VF mean deviation [MD] ≥ −12 dB) and severe glaucoma (VF MD < −12 dB).

The normal controls showed intraocular pressure (IOP) less than or equal to 21 mm Hg, had no history of IOP elevation, no glaucomatous optic disc appearance, no RNFL defect, and normal HVF results. Normal HVF results were defined as an MD and pattern standard deviation within the 95% confidence limits and a glaucoma hemifield test result within the normal limits. If both eyes were eligible, one was selected randomly.

### Imaging of outer retinal layer

**Figure 5:**
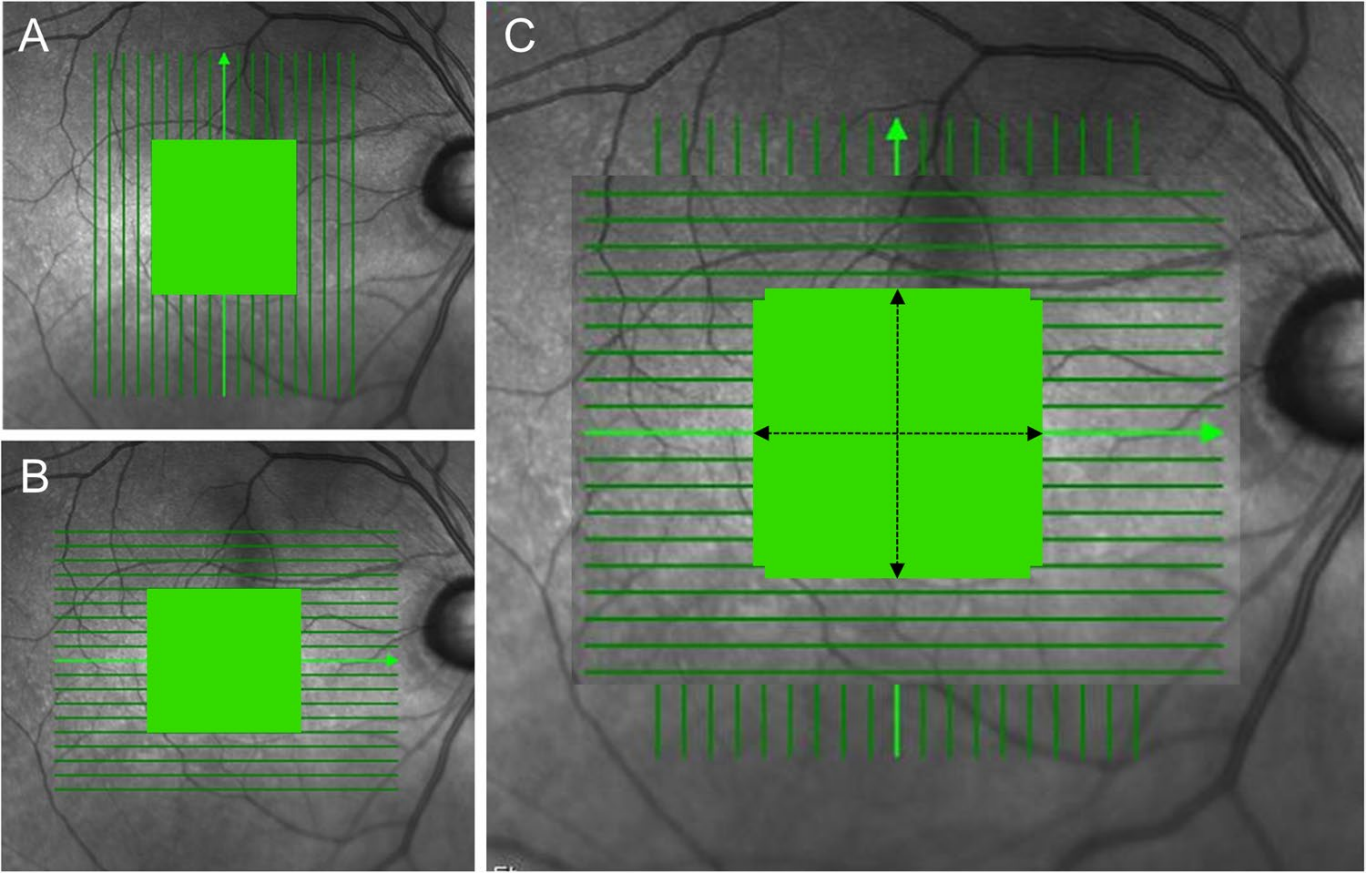
Spectral-domain OCT macular scan area. (**A**) Nineteen (19) vertical and (**B**) 19 horizontal line scans (length: 9000 µm). (**C**) Demarcation of total analyzed area (length: 4000 µm) for 11 horizontal along with 11 vertical retinal scans (green color).

All of the subjects underwent SD-OCT confocal scanning laser ophthalmoscopy (Spectralis HRA + OCT; Heidelberg Engineering, V Heidelberg, Germany) using the eye-tracking feature (TruTrack; Heidelberg Engineering, Heidelberg, Germany). All of the images were obtained through dilated pupils by a single experienced examiner. The 19 horizontal and 19 vertical line scans of 9-mm length were obtained in the high-resolution setting, and 128 frames were averaged (Fig. 5). Presence of foveal bulge, foveal depression, and inner-retinal-layer thinning, all on SD-OCT, was considered to confirm the foveal area. For inclusion, all of the images were reviewed for non-centered scans or artifacts, and all had a signal quality >20 dB.

### Manual mEZi quantification method

The details of the methodology for manual quantification of mEZi have already been reported^16^. Briefly, mEZi intensity was determined as the ratio of the second reflective band to the first (i.e., the EZ/ELM [external limiting membrane] ratio) to account for the variation of OCT scan brightness. Logarithmic-transformed B-scans of each eye of each participant were rendered in the tagged image file format (TIFF).

**Figure 6:**
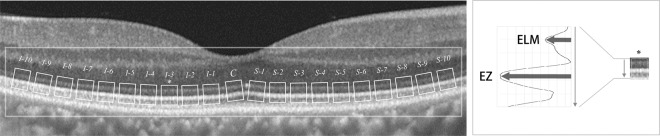
Illustrative diagram of 21 ellipsoid zone (EZ) retinal locations and sampling of external limiting membrane (ELM) at fovea and to maximum of 2000 µm eccentricity from fovea, vertically. Each sample was 150 μm wide and had intervals of 200 μm. Accordingly, an EZ segment’s “relative intensity” was calculated by dividing the EZ segment intensity by the ELM intensity. The retinal segment (I-3) that was used for the relative EZ intensity calculation is marked by an asterisk.

EZ is known to be less distinct according to eccentricity^17^. For the purposes of consistent and accurate mEZi measurements, then, we analyzed the central macular area (total 4000 µm-length) only. The mEZi (4000-µm of 11 horizontal and 11 vertical retinal scans) was averaged, each meridian comprising 21 retinal segments. The mEZi was measured based on the highest EZ band intensity value as divided by the highest ELM band intensity value of relevant SD-OCT imaging (Fig. 6). All measurements were performed using the public-domain NIH Image program (ImageJ 1.48 v, Wayne Rasband, National Institutes of Health, Bethesda, MD, USA). To avoid retinal vessels’ local “shadowing” effects on EZ and ELM intensity, any such segments were excluded from further analysis. Experienced ophthalmologists (YKK and AH) who were masked to the patients’ clinical information performed all of the intensity measurements, independently. The average value of the two graders’ measurements was used as the final MM mEZi. The results of the inter-observer comparison are presented as Supplementary Fig. S2.

### Automated mEZi quantification

**Figure 7:**
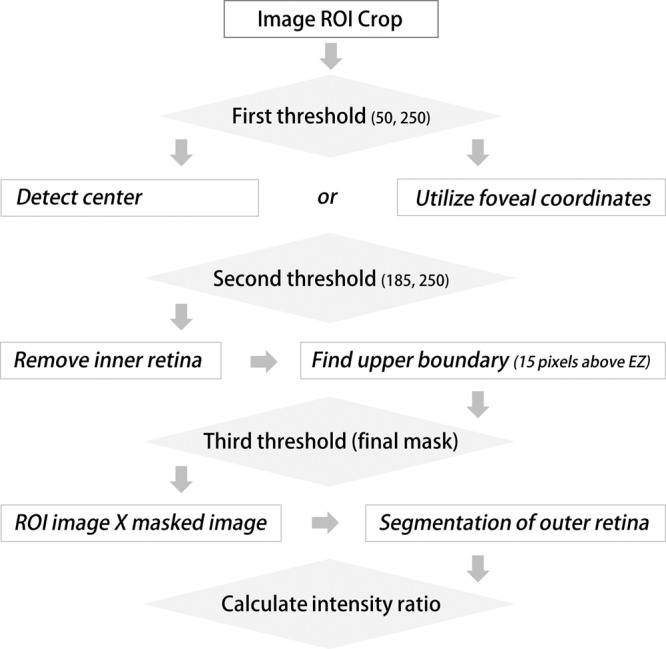
Flowchart of the automated macular EZ intensity (mEZi) quantification method. By thresholding processes, the software eliminates the inner retinal layer and extracts the outer retinal layers including the ELM and EZ. The mEZi was determined by calculating the ratio of the EZ/ELM intensity peaks.

The AM was designed to provide an average mEZi (i.e., the EZ/ELM ratio). In each line scan, the mEZi was quantified in 220 evenly spaced sectors. Therefore, in each eye, the mEZi was quantified in 4,420 sectors (220 sectors x 11 lines) at both the horizontal and vertical meridians. The AM, based on Python programming, utilized Opencv library for image processing. For details on the algorithm used in developing the AM, see Fig. 7 and Appendix A.

### Calibration study - Determining relationship of AM to MM

To compare AM with MM mEZi quantification for different mEZi ranges, OCT images were obtained from the three representative disease-stage groups: normal (*n* = 40), mild-to-moderate glaucoma (*n* = 60), and severe glaucoma (*n* = 60). The output data for each image set was the mEZi as quantified by the AM and MM, and the results were directly compared. The relation between the AM and MM quantifications was assessed by Deming regression. A Bland-Altman plot was generated to assess the inter-method agreement.

### Validation study - comparison of compensated AM with MM quantification

For algorithm validation, AM mEZi quantification was compared with both the mean and range of repeatability (95% CI) as derived from five separate and repeated MM mEZi quantifications. In this validation study, a separate group of 33 representative images was employed. This group’s images represented the normal (*n* = 11), mild-to-moderate glaucoma (*n* = 11), and severe glaucoma (*n* = 11) eyes. The MM mEZi quantification was performed on all of the 33 images sets by the same operator (YKK) at five separate times separated by at least 3 days. The correlation between AM and MM quantification was assessed by Deming regression, as outlined above. The AM quantification for each image set was then compensated by using the equation resulting from the linear regression of the difference versus average mEZi quantification for the calibration set. The number of images for which compensated AM quantification was outside of the 95% CI of MM quantification was recorded.

### Data analysis

Comparison of mEZi distribution in each method (i.e. calibration and validation) was performed by Mann-Whitney test. Inter-method and inter-observer comparisons were performed using Deming regression and Bland-Altman plots. Deming regression is an errors-in-variables model that finds the line offering the best fit for a two-dimensional dataset^18^. It differs from simple linear regression in that it calculates for observation errors on both the x- and y- axes. Deming regression assesses inter-method linearity according to statistical significance based on the CI for the slope not containing 1 and the CI for the intercept not including 0^18^. Bland‐Altman plots are presented below for visual representation of the inter-method agreement. Statistical analysis was performed using the MedCalc software (version 12.1.3.0, Mariakerke, Belgium); a *P* value less than 0.05 was considered statistically significant.

### Supplementary information

Supplementary Information

Dataset 1

## Data Availability

The dataset generated during the current study is available in the Supplementary Material.

## Appendix A

The algorithm runs as follows:

1. Obtain regions of interest (ROI) image: read image and obtain x, y coordinates. Crop macular image according to pixel numbers to include central area of 4000-µm length.
2. First thresholding for creation of binary image map: pixels between 50 and 250 are replaced with 255 to find foveal center location.
3. Detection of foveal center by measuring of longest distance from upper margin of image. For images outside fovea, center coordinates obtained from foveal image are applied.
4. Second thresholding for elimination of inner retinal structures: pixels between 185 and 250 are replaced with 0. This process leaves only three bright layers (i.e., EZ, interdigitation zone and retinal pigment epithelium) in outer retinal layer.
5. Define region above 15 pixels from EZ as upper boundary to include ELM.
6. Third thresholding for restoration of original outer retinal structures: all pixels of 255 are converted to 1.
7. Multiplication of ROI image by this binary masked image to extract outer retinal layers including ELM and EZ.
8. Finally, determine mEZi by calculating ratio of intensity peaks by EZ and ELM.
9. Exclude upper and lower 5% values (due possibly to artifacts such as vessel shadowing) from final analysis.
