# Supplementary material for: Automated Quantification of Macular Ellipsoid Zone Intensity in Glaucoma Patients: the Method and its Comparison with Manual Quantification
Source: Sci Rep. 2019 Dec 24;9:19771. doi: 10.1038/s41598-019-56337-7 (PMC6930206; doi:10.1038/s41598-019-56337-7)
Supplement: Supplementary file 1 — Supplementary Information [file 41598_2019_56337_MOESM1_ESM.docx]

**Automated Quantification of Macular Ellipsoid Zone Intensity in Glaucoma Patients:**

the Method and its Comparison with Manual Quantification

Ahnul Ha, MD, Sukkyu Sun, MS, Young Kook Kim, MD, Jin Wook Jeoung, MD, PhD, Hee Chan Kim, PhD, Ki Ho Park, MD, PhD

**Figure S1. Comparison of automated method (AM) versus manual method (MM) mEZi quantification for different stages of glaucoma.** (A) AM versus MM of mEZi quantification, with 120 images for calibration. (B) AM versus MM of mEZi quantification, with 22 images for validation. The blue dots indicate mild-to-moderate glaucoma patients, and the red dots represent severe glaucoma patients. (C) Bland-Altman plot for validation images in mild-to-moderate glaucoma patients. (D) Bland-Altman plot for validation images in severe glaucoma patients.

**
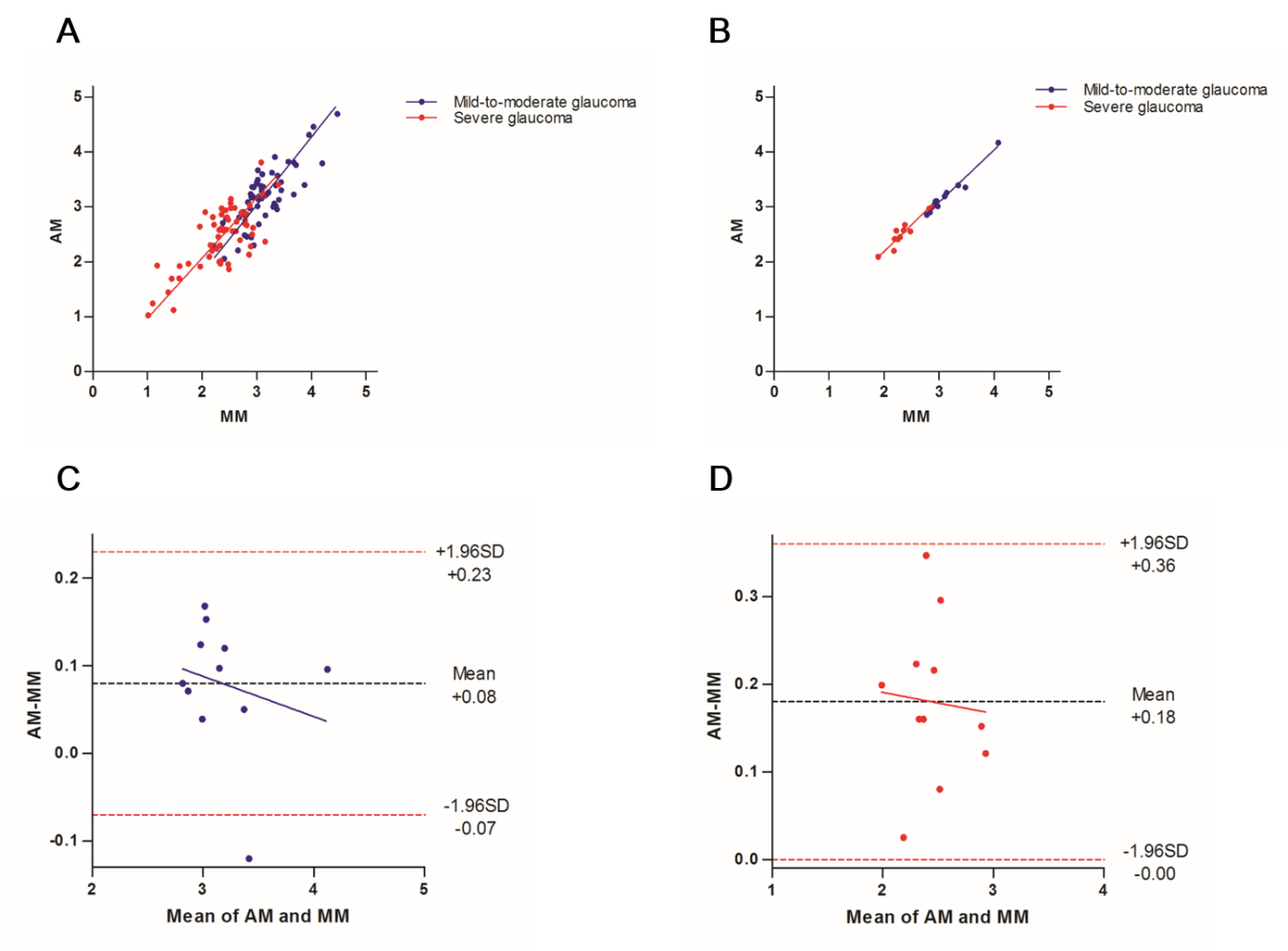
**

**Figure S2. Inter-observer comparison for MM of mEZi quantification.** (A) Two experienced ophthalmologists performed the measurements (MM_1_ by YKK and MM_2_ by AH). The relation of MM_1_-to-MM_2_ quantification, as established by Deming regression, is defined by the solid line according to the formula MM_2_ = 0.961(MM_1_) + 0.105. (B) Bland-Altman plot for 160 calibration images. The dashed red lines represent the 95% limits of agreement between the MM_1_ and MM_2_ (-0.82 and +0.86).

**
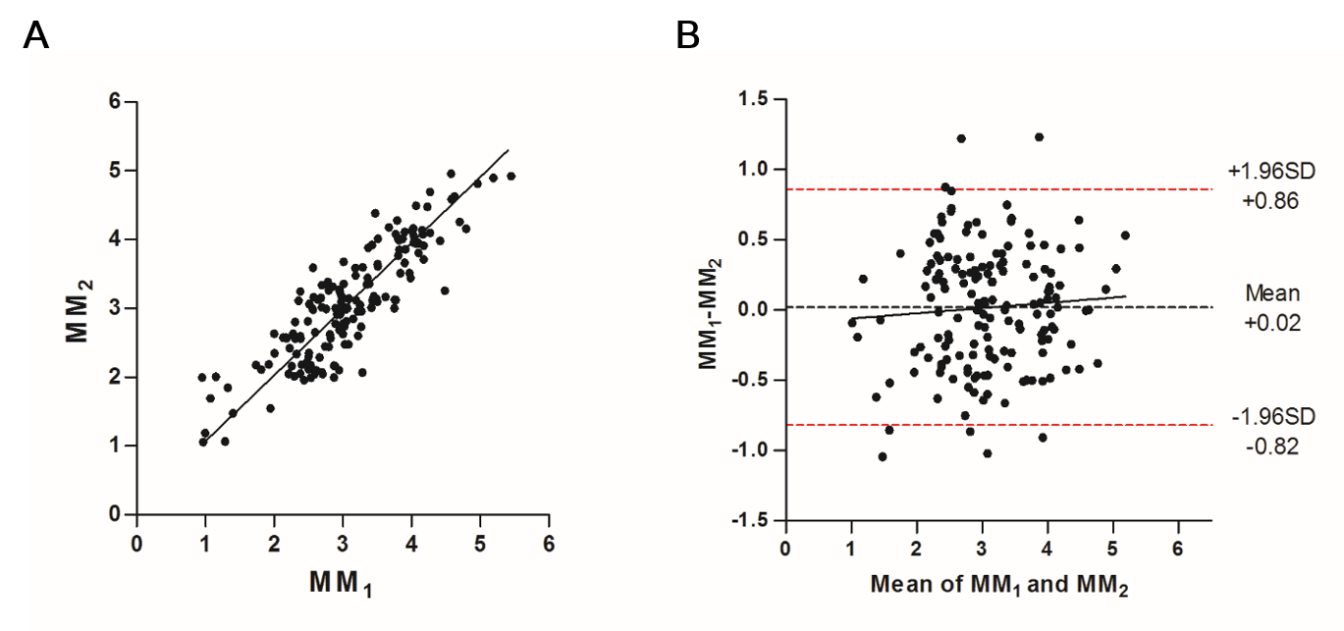
**
